# Modeling and Preventive Measures of Hand, Foot and Mouth Disease (HFMD) in China

**DOI:** 10.3390/ijerph110303108

**Published:** 2014-03-13

**Authors:** Yong Li, Jinhui Zhang, Xinan Zhang

**Affiliations:** School of Mathematics and Statistics, Huazhong Normal University, No. 152 Luo Yu Road, Wuhan 430079, China; E-Mails: gxbyl@163.com (Y.L.); jinhuitao20@126.com (J.Z.)

**Keywords:** HFMD, the basic reproductive number, Chi-square test, goodness of fit

## Abstract

This paper concentrates on the HFMD data of China from March 2009 to December 2012. We set up a mathematical model to fit those data with the goodness of fit and obtain the optimal parameter values of the model. By the Chi-square test of statistical inference, the optimal parameter values of the model are reasonable. We obtained the basic reproductive number of the disease for each year, and it is larger than 1. Thus, we conclude that HFMD will persist in China under the current conditions, so we investigate the preventive measures to control the HFMD. If the preventive measures proposed in our paper were implemented, HFMD would be controlled quickly and the number of infections would decline rapidly over a period of time.

## 1. Introduction

Hand, foot and mouth disease (HFMD), an infectious disease caused by enterovirus and Coxsackievirus, usually happens to children under age of five, with an especially high incidence being observed for those under three. It can result in herpes in such body parts as hands, feet and mouth and even other complications such as myocarditis, pulmonary edema, and aseptic meningoencephalitis in some children. Some severely affected patients may die due to the quick progress of the disease [1]. There are over twenty types of enterovirus leading to HFMD. The common pathogens of HFMD are Type 16, Type 4, Type 5, Type 9, and Type 10 for Coxsackievirus Group A, Type 2 and Type 5 for Coxsackievirus Group B, and Enterovirus 71 (EV71). Coxsackievirus A16 and EV71 are the most common, and cause severe mortality in children.

HFMD spreads to children in the following ways: close contacts with crowds, touching virus-carrying hands, towels, handkerchiefs, toys, utensils, bedding and underclothes and alike, air-borne spread and mouth-to-mouth spread of the virus in the throat secretions and saliva, and consuming infected water and food. Adults are immune to the disease due to the antibodies in their bodies although most of them are exposed to the virus. Some 80% of the infected are children under the age of five. Most adults do not have any symptoms after infection, which is called a latent infective status. Most infants and very few adults would present symptoms, which is called apparent infective status. The proportion of latent to apparent infections is about 100:1. Most infected children can be cured without any sequela after infection. Ulcerative stomatitis will heal up in a week or so, and the herpes in vola will recover in ten days or so. However, delayed diagnoses and treatment may cause heart, brain and kidney complications that can result in the death of the infected children. HFMD is a highly contagious disease with a latency period of 3–7 days. The possibility of serious symptoms caused by the virus infection by HFMD is much higher than that by other types of enterovirus [2].

In 1957, the first case of HFMD was reported in New Zealand. In 1958, the Cox Virus was extracted and purified in Canada and the disease which caused by Cox Virus was named HFMD in America in 1959. Enterovirus 71 which was later realized as the other kind of HFMD virus was first discovered in California [3] and then outbreaks of HFMD epidemic ocurred in many areas of the world, such as the United States of America, Australia, Europe, Brazil, Japan, and Malaysia [3,4,5,6,7,8,9,10,11]. These outbreaks of HFMD have caused panic among people from all over the world, making people worry about such epidemics of HFMD, because innumerable people were infected and many of them were in immediate danger of death and even died eventually. In 1975, an epidemic of HFMD appeared in Bulgaria, 750 people were infected, 149 people had paralyses and 44 people died [5]. In 1997, a HFMD epidemic attacked Sarawak, Malaysia, causing 34 children deaths [11]. In 1998, a large-scale epidemic of HFMD occurred in Taipei, where according to the statistical data of the local government, there were 129,106 people who were infected by HFMD virus or herpangina, and 405 people got even worse, with neurological complications or pulmonary edema, and finally 78 children died during this HFMD epidemic [12,13,14,15,16]. In China, there have also been reports of HFMD cases such as an epidemic of HFMD that broke out in Fuyang, Anhui Province in 2008, where in a short period, there were 25,000 people infected with HFMD virus, and among these, 42 children lost their lives [17]. In 2010, an outbreak occurred in southern China’s Guangxi Autonomous Region as well as in Guangdong, Henan, Hebei and Shandong provinces. Until March 70,756 children were infected and 40 children died from the disease. By June, 537 children had died [18]. Besides, in other places or countries of Asia, outbreaks of HFMD epidemic have also occurred and been publically reported.

Nowadays, people have become aware that HFMD is dangerous to the health of human beings, especially to infants and children and that it is a threat to society. Tiing and Labadin used a deterministic SIR model to predict the sum of infected people and the duration of an outbreak as it occured in Sarawak [19]. Liu used a SEIQRS models to simulate the dynamics of HFMD transmission, got a positive periodic solution, and proved that the disease persists [1]. Su and Liu set up an age-structured HFMD model with isolation to analyze the conditions of the existence of the endemic equilibrium [20]. Ma *et al.* proposed a dynamic model with periodic transmission rates to investigate the seasonal HFMD [2].

This paper is organized as follows: Section 2 gives the data of HFMD in China and studies how to build a suitable epidemic model. Section 3 shows the results. Section 4 presents the corresponding conclusions and possible preventive measures to control HFMD.

## 2. Methods

### 2.1. Data

The Ministry of Health of the People’s Republic of China declared that HFMD was ranked as a Class C Infectious Disease on May 2nd, 2008. The Chinese Center for Disease Control and Prevention (China CDC) does statistical work on the number of people who have HFMD every month [21]. The following is the data from China CDC from March 2009 to December 2012 in China (Hong Kong, Macao and Taiwan are not included in this work.). See Table 1 (where “– –” indicates missing data). 

In general, medical staffs easily determine HFMD infections just by relying on symptoms such as a slight fever followed by blisters and ulcers in the mouth and rashes on the hands and feet. In fact, it is easy to distinguish between chicken pox, dental ulcer, foot-and-mouth disease, herpangina, scarlet fever and so on. Furthermore, the final result of the diagnosis will be made in the laboratory according to samples of throat swabs or feces. So the data from the China CDC comprises the reported data of HFMD from one place or another, and clinically confirmed cases. Once an outpatient is examined to confirm HFMD infection, he or she would be necessarily be hospitalized, so the data that China CDC provides covers hospitalization. Certainly, we have to ignore the rare cases where the patients’ conditions were relatively mild and they returned home for treatment with the doctors’ permission.

**Table 1:** The number of the HFMD patients reported every month/year.

Mouth/Year	2009	2010	2011	2012
January	– –	37,567	32,179	50,758
February	– –	23,862	10,609	40,505
March	54,713	77,756	34,709	99,052
April	212,435	248,609	99,819	237,478
May	169,073	354,347	230,460	462,116
June	178,680	343,100	303,594	381,626
July	162,060	261,263	253,442	248,739
August	99,897	119,096	132,154	118,333
September	85,504	101,654	120,802	135,974
October	76,948	87,612	122,491	146,392
November	61,918	79,591	152,768	150,264
December	47,817	60,879	126,679	127,205

### 2.2. Model Analysis

**Figure 1:**
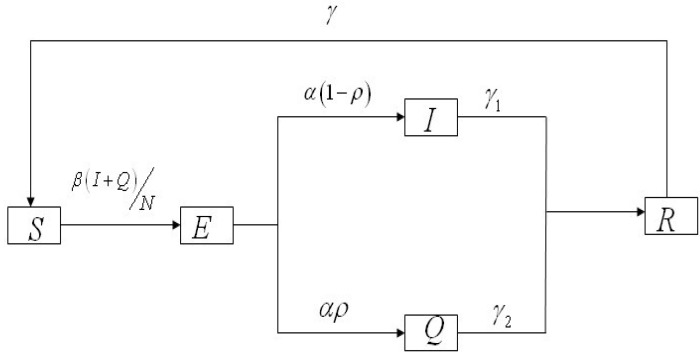
Flow chart of compartments of the HFMD model.

The population associated with HFMD is divided into five compartments: The susceptible (*S*), exposed (*E*), infectious and not hospitalized (*I*), infectious and hospitalized (*Q*), and recovered (*R*) individuals. The total population is *N* = *S* + *E* + *I* + *Q* + *R*. We use *β*, *α*, *ρ*, *γ*_1_, *γ*_2_, *γ* to denote the transmission rate, the rate of progression to the infectious and not hospitalized, the proportion of the infectious and hospitalized, the recovery rate of the infectious and not hospitalized, the recovery rate of the infectious and hospitalized individuals, the ratios of the recovered individuals becoming susceptible individuals (Figure 1).

### 2.3. Parameters and Model Hypothesis

(1) Given the short course of HFMD, it is reasonable that we ignore the mobility of the patients, the space structure and the environmental climate of the model to collect all these data.

(2) According to the annual data from the China CDC, we can assume the initial numbers *Q*(0) (see Table 2.).

(3) We fix the birth and death rate *µ* = 1/(70 × 365) = 3.9139 × 10^−5^. We select 10% of the total population in China as the object of our investigation, that is *S*(0) = 1.4 × 10^8^.

(4) By simulation, we find that the mortality rate *δ* of HFMD is very small and almost equal to 0, that is *δ* = 0. 

(5) According to the biological significance, we set the range of each parameter reasonably in Table 2.

### 2.4. Model Formulation

The differential equations for HFMD model are:

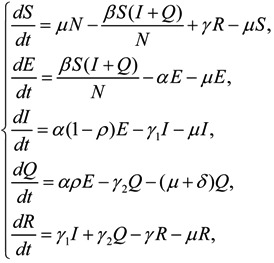
(1)
with initial conditions:

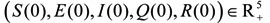
(2)


Following van den Driessche and Watmough [22], we can compute the basic reproduction number:

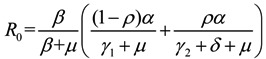
(3)


## 3. Results

### 3.1. Parameter Estimation

We estimate parameters of system (1) by calculating the minimum sum of square (MSS) [23]:

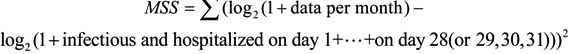
(4)
with the MATLAB (The Mathworks, Inc., Natick, MA, USA) tool fminsearch, which is a part of optimization toolbox. All optimal parameter values are obtained only when the results of fminsearch are convergent. Those values are shown in Table 2. Here, we also calculate the basic reproduction number.

**Table 2:** Estimation of parameters and the basic reproduction number from 2009 to 2012.

Parameter Interval and *R*_0_	Source	2009	2010	2011	2012
*β* in [0.0001,50]	MSS	1.0252	0.5575	0.5149	0.7738
*γ*_1_ in [0.1,1]	MSS	0.9419	0.5136	0.4744	0.7348
*γ*_2_ in [0.1,1]	MSS	0.4034	0.2251	0.3080	0.2013
*α* in [0.0001,1]	MSS	0.3907	1	1	1
*ρ* in [0.01,1]	MSS	0.01	0.01	0.01	0.01
*γ* in [0.0001,1]	MSS	0.0089	0.0072	0.0126	0.0111
*S*(0)	Fixed	1.4 × 10^8^	1.4×10^8^	1.4 × 10^8^	1.4 × 10^8^
*E*(0)in [1.8 × 104,1.4 × 107]	MSS	2.4029 × 10^5^	2.0430 × 10^4^	1.8290 × 10^4^	1.9790 × 10^4^
*I*(0)in [1.8 × 103,1.8 × 105]	MSS	1.9469 × 10^3^	1.8001 × 10^3^	1.8 × 10^3^	1.8001 × 10^3^
*Q*(0)	Fixed	1,765	1,212	1,038	1,673
*R*(0)	Fixed	0	0	0	0
*R*_0_	Calculated	1.1028	1.0993	1.0911	1.0809

It is shown that the same parameters in the model (1) change relatively little in every year in Table 2, so we make predictions about the prevalence trends in subsequent years with the help of the data of HFMD from 2009 to 2012 in China, which means that our research work possesses a certain reference value. More specifically, by analyzing the parameters *α*, *γ*_2_, *R*_0_ in Table 2 we draw up the following conclusions conformed to reality that the incubation period 1/*α* is approximately equal to 1–2.5595 days, and the course of treatment 1/*γ*_2_ is about 2.4786–4.9677 days. Moreover, *R*_0_, indicating the numbers of persons that one patient infects in an average sick period, is basically stable at around 1.1, which explains why HFMD has yet not broken out on a large scale despite the fact that it continues to be prevalent in China.

### 3.2. Chi-Square Test of Goodness of Fit

In order to test how well our model reflects the data actually, we consider the following hypotheses:

Null hypothesis, *H*_0_: The estimated parameters are equal to actual values.

Alternative hypothesis, *H*_1_: The estimated parameters are not equal to actual values.

The Chi-square values and degrees of freedom for each year are shown in Table 3. Therefore, we cannot reject the null hypothesis at the 5% significant level by Pearson's criterion of Chi-square test [24].

**Table 3:** Chi-square values and degrees of freedom for each year.

	2009	2010	2011	2012
Chi-square value	0.0525	0.1094	0.257	0.1012
*υ* *	1	3	3	3
AV *	3.841	7.815	7.815	7.815

Note: * *υ* denotes degrees of freedom. * AV denotes the accepting value at 5% significant level with degrees of freedom 1 or 3.

**Figure 2:**
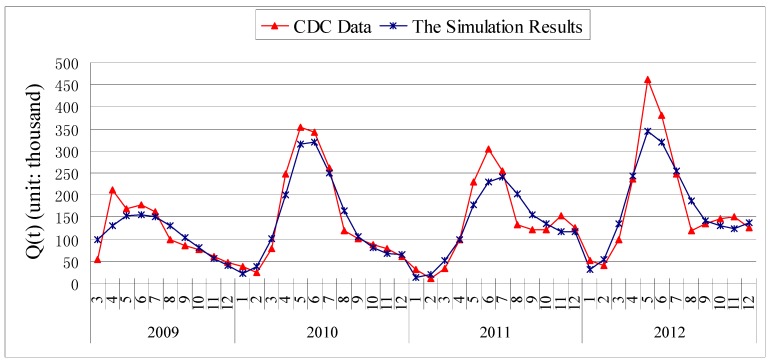
The comparison chart of the data of HFMD in China and simulation results by model (1).

The numerical simulation of the number of the infectious and hospitalized individuals of HFMD by model (1) is shown in Figure 2. The simulation provides a good match with the data of HFMD in China from March 2009 to December 2012.

### 3.3. Sensitivity Analysis

Next, we consider the impact of parameters on *R*_0_. Considering *β*, *ρ*, *γ*_1_ and *γ*_2_ as an independent variable and the other parameters as constants, respectively. One obtains implicit differentiation of *R*_0_ with respect to *β*, *ρ*, *γ*_1_ and *γ*_2_, respectively:

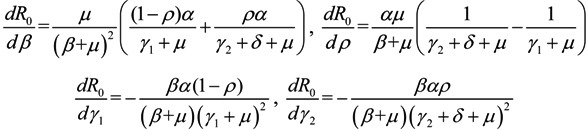
(5)


By the signs of 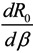, 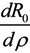, 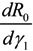
and 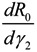, we can see that *R*_0_ decreases as *β* decreases, *R*_0_ decreases as *γ*_1_ or *γ*_2_ increases, and the impact of *ρ* on *R*_0_ depends on the values of *γ*_1_ and *γ*_2_. In theory, if we can control some parameters such that *R*_0 _< 1, then the disease will die out. On this basis we shall put forward detail preventive strategies of HFMD in the following section.

## 4. Discussion and Conclusions

There have been plenty of papers on how to control and prevent HFMD from a public health and statistical model perspective. However, there are few works [2] constructing differential equations models to simulate data of HFMD. All the parameters of the model proposed in [1] and [2] are estimated without using statistical methods to verify the rationality of the parameters. Consequently, we demonstrate rationality of the parameters in our model as well as consistence with reality by applying the Chi-square test.

In this paper, we consider the HFMD data reported by China CDC, and construct a SEIQRS model to fit the HFMD data. From the last column in Table 2, the basic reproductive number in each year is larger than 1. Thus, we conclude that HFMD will persist in China under the current conditions. As a matter of fact, there is no effective vaccine or antiviral treatment specifically for HFMD, but if we can provide some preventive measures to control the HFMD, it will be very meaningful. 

**Figure 3:**
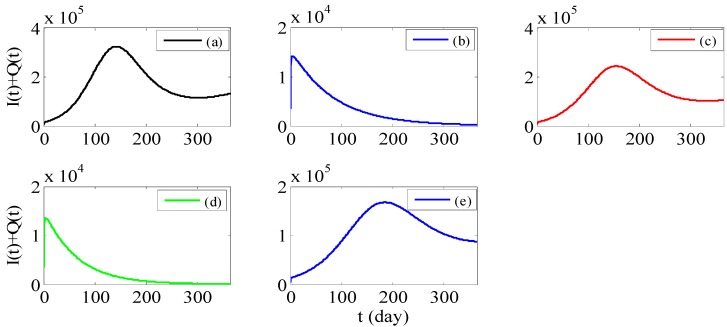
(**a**) Simulation of the sum of not hospitalized infectious *I(t)* and hospitalized infectious *Q(t)* with parameters from the sixth column of Table 2. (**b**) Simulation of the sum of *I(t)* and *Q(t)* with *β* = 0.6964 (=0.7738 × 0.9), other parameters from the sixth column of Table. (**c**) Simulation of the sum of *I(t)* and *Q(t)* with *ρ* = 0.005, other parameters from the sixth column of Table 2. (**d**) Simulation of the sum of *I(t)* and *Q(t)* with *γ*_1_ = 0.8267 (=0.7348 × 1.125), other parameters from the sixth column of Table 2. (**e**) Simulation of the sum of *I(t)* and *Q(t)* with *γ*_2_ = 0.8052 (=0.2013 × 4), other parameters from the sixth column of Table 2.

Next, we select the year of 2012 as an example (The simulation of the sum of not hospitalized infectious *I(t)* and hospitalized infectious *Q(t)* is presented in Figure 3a.), and propose some preventive measures as follows: 

Strategy 1: Reducing the transmission rate *β* for the susceptible can effectively control the spread of HFMD (see Figure 3b). Therefore, health-care education such as washing hands before meals and after using the toilet, and making air fresh indoors and so on, should be carried out in kindergardens, schools, hospitals and other places to popularize health knowledge and advocate good personal hygiene habits. Kindergardens should clean and disinfect toys and appliances every day. In addition, hospitals should strengthen infection control practices to avoid nosocomial cross infection.

Strategy 2: Reducing the rate of progression to infective individuals *ρ* can control the spread of HFMD at a lower level (see Figure 3c). For example, more fruits, vegetables, and regular exercise will increase their immunity. Even if they carry the enterovirus, they may not be infected.

Strategy 3: Increasing the recovery rate of non-hospitalized infectious individuals *γ*_1_ can effectively control the spread of HFMD (see Figure 3d), so we suggest they should see a doctor in a timely fashion and thus reduce the chance of contact with other people when the adults appear to have symptoms of fever, rash and so on. However, changing the recovery rate of hospitalized infectious individuals *γ*_2_, the control effect is relatively small (see Figure 3e).

In a word, if we use the above preventive measures, the HFMD will be controlled quickly and the number of infections will decline rapidly in a period of time. Those measures can effectively prevent the large-scale diffusion of the disease.

We can see that *β* and *γ*_1_ are the most sensitive parameters comparing Figure 3b,d with the others because just slight changes can achieve the goal of control. These existing measures to control and prevent HFMD can be essentially attributed to how to reduce *β*. Based on the discussion in this paper, it is vitally important not only to reduce *β* but also to increase *γ*_1_. In addition, it is more effectively to increase *γ*_1_ than to increase *γ*_2_ precisely because persons with latent infection are more than apparent infection. So far, there are few papers using ordinary differential equations models to simulate the real data of HFMD and make measures to control and prevent it. Not only that, a powerful theoretical basis is provided for more detail control countermeasures in our paper.
